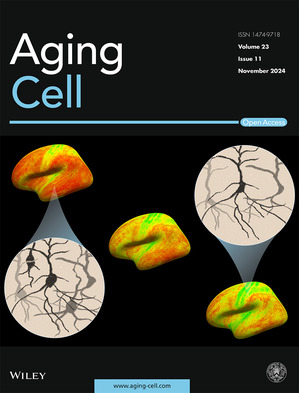# Featured Cover

**DOI:** 10.1111/acel.14417

**Published:** 2024-11-14

**Authors:** Hansol Lee, Hong‐Hsi Lee, Yixin Ma, Laleh Eskandarian, Kyla Gaudet, Qiyuan Tian, Eva A. Krijnen, Andrew W. Russo, David H. Salat, Eric C. Klawiter, Susie Y. Huang

## Abstract

Cover legend: The cover image is based on the Article *Age‐related alterations in human cortical microstructure across the lifespan: Insights from high‐gradient diffusion MRI* by Hansol Lee et al., https://doi.org/10.1111/acel.14267